# Editorial: Evolution of antimicrobial resistance in intracellular bacterial pathogens: a genomic and historical perspective

**DOI:** 10.3389/fmicb.2026.1995224

**Published:** 2026-09-25

**Authors:** Kumaragurubaran Karthik, Maryam Dadar, Subbaiyan Anbazhagan, Axel Cloeckaert

**Affiliations:** 1 Veterinary College and Research Institute, Veterinary and Animal Sciences University, Udumalpet, Tamil Nadu, India; 2 Department of Brucellosis, Razi Vaccine and Serum Research Institute (RVSRI), Agricultural Research, Education and Extension Organization (AREEO), Karaj, Iran; 3 Veterinary Council of Iran, Tehran, Iran; 4 ICMR-National Institute for Pre-Clinical Research, Hyderabad, India; 5 INRSAE, Université de Tours, UMR, ISP, Nouzilly, France

**Keywords:** antimicrobial resistance, *Bartonella*, *Burkholderia*, comparative genomics, host–pathogen interactions, intracellular pathogens, *Mycobacterium tuberculosis*, resistome

Intracellular bacterial pathogens including *Mycobacterium tuberculosis, Brucella* spp., *Listeria* spp., *Burkholderia* spp., and *Coxiella burnetii* pose severe therapeutic challenges. Their capacity to survive and replicate within host cells limits the efficacy of many conventional antimicrobials, which frequently fail to achieve adequate intracellular concentrations or are compromised by acquired resistance mechanisms. The rapid global rise of antimicrobial resistance (AMR) among both extracellular and intracellular pathogens underscores an urgent need for deeper insight into the evolutionary trajectories of resistance. Comparative genomics, mutational analyses, and phylogenetic reconstruction offer powerful tools to decipher how resistance determinants emerge and disseminate during host infection.

The main objective of this Research Topic was to trace the historical development and genomic drivers of AMR in intracellular pathogens during human and animal infections, focusing on key species such as *Brucella melitensis, Listeria monocytogenes, Mycobacterium bovis, Mycobacterium orygis, Burkholderia mallei*, and *Burkholderia pseudomallei*. The six articles published within this topic collectively illuminate complementary facets of resistance emergence, persistence, and adaptation in these organisms.

In *M. tuberculosis* (Mtb), phosphoserine phosphatase SerB2 has been recognized as a promising therapeutic target, yet the precise relationship between its structural dynamics and enzymatic function has remained unclear. Liu et al. evaluated the hydrodynamic properties of SerB2 and created site-specific mutants, demonstrating that amino acid residues 294–314 impart significant structural flexibility compared to other domains. Notably, their screening identified clofazimine as a potent inhibitor of SerB2 among the

tested compounds. This study provides fundamental structural and functional insights into SerB2 while shedding light on the molecular mechanisms underlying clofazimine's anti-tubercular activity.

Species within the genus *Bartonella* are facultative intracellular pathogens responsible for clinical conditions such as cat-scratch disease (*Bartonella henselae*), Carrion's disease (*Bartonella bacilliformis*), and trench fever (*Bartonella quintana*). Although bartonellosis can present as a self-limiting illness, it is frequently life-threatening in immunocompromised patients. Given the lack of a standardized treatment protocol to effectively clear intracellular *Bartonella* infections, Olsen and Embers investigated combination antibiotic regimens. Their findings revealed that while monotherapy effectively targets extracellular bacterial stages, combining gentamicin with either rifampin or azlocillin is necessary to significantly reduce intracellular bacterial loads.

Intracellular bacterial adaptation involves dynamic processes such as genome reduction, horizontal gene transfer, altered transport systems, stress-response regulation, and mutations affecting drug targets, membrane permeability, and efflux pumps. Metabolic flexibility is equally vital: pathogens dynamically switch carbon sources, scavenge host lipids or amino acids, remodel host cellular metabolism, and enter slow-growing or dormant states. These phenotypic adaptations give rise to antibiotic tolerance and persistence, establishing reservoirs from which genetically resistant sub-populations can subsequently emerge. In their comprehensive review, Nair et al. emphasize that AMR should be understood as a dynamic evolutionary interaction among the pathogen, host, and therapeutic selective pressure, rather than merely an isolated bacterial genetic defect. The authors argue that future interventions must integrate genomics, metabolomics, and single-cell analyses to target vulnerable regulatory and metabolic networks. Host-directed therapies deployed alongside conventional antibiotics may enhance intracellular drug delivery and attenuate selective pressure, offering a promising strategy to combat persistent and drug-resistant infections.

The empirical study by Jaleta et al. provides critical insights into the dynamics of antimicrobial resistance genes (ARGs) within livestock production, offering a valuable lens for understanding the broad genomic evolution of bacterial resistomes. Utilizing high-throughput qPCR (SmartChip) and targeted quantitative PCR, the authors identified high intra-cohort variability in ARG carriage among weaned pigs despite uniform management and environmental conditions. Key determinants such as *erm*(B), *aadA1*, and *tet*(A) dominated the swine resistome, whereas other genes, such as *qnrS*, exhibited limited host-to-host transmission. From a historical and genomic perspective, these findings highlight that intracellular adaptation and ARG dissemination are heavily shaped by host microenvironments, gut maturity, and environmental reservoirs, rather than by direct antimicrobial exposure alone. Consequently, effective One Health surveillance demands routine monitoring of livestock environments paired with targeted dietary interventions to stabilize the microbiome and curb resistance dissemination.

The withdrawal of official Clinical and Laboratory Standards Institute (CLSI) and European Committee on Antimicrobial Susceptibility Testing (EUCAST) clinical breakpoints for the *Burkholderia cepacia* complex (Bcc) has left healthcare providers navigating a clinical vacuum. Rajni et al. highlight that the absence of standardized interpretive criteria (susceptible/intermediate/resistant) introduces significant clinical uncertainty when selecting therapeutic regimens for these highly resistant opportunistic pathogens. Reporting raw minimum inhibitory concentration (MIC) values or relying on non-species-specific epidemiological cut-off values (ECOFFs) risks clinical misinterpretation and treatment failure. Bridging this critical gap requires strengthened diagnostic-clinical collaboration, rigorous multicenter studies linking MIC data with patient outcomes, and updated antimicrobial stewardship protocols to manage Bcc infections safely in the absence of traditional guidelines.

Abdulai et al. conducted a cross-sectional abattoir-based study to understand the ovine pneumonic pathogens. *Pasteurella multocida* was the major pathogen in interstitial pneumonia cases and the isolates were susceptible to cefotaxime and ciprofloxacin while resistant to amoxicillin and doxycycline.

In conclusion, the contributions to this Research Topic advance our understanding of how intracellular bacterial pathogens acquire, maintain, and disseminate resistance mechanisms. The studies in this editorial illuminate the problem of AMR in intracellular bacterial pathogens from complementary angles, although deep evolutionary and phylogenetic analyses remain limited. Nevertheless, existing data remain insufficient to draw definitive conclusions regarding the long-term evolutionary trajectories of AMR in these complex pathogens. Large-scale, longitudinal genomic and functional studies integrating clinical, veterinary, and environmental sampling will be essential to map resistance dynamics comprehensively and translate these mechanistic insights into improved therapeutic and surveillance strategies.

## Generative AI statement

The author(s) declared that Generative AI was not used in the creation of this manuscript.

Any alternative text (alt text) provided alongside figures in this article has been generated by Frontiers with the support of artificial intelligence and reasonable efforts have been made to ensure accuracy, including review by the authors wherever possible. If you identify any issues, please contact us.

